# Time Trends and Regional Variation in Prevalence of Asthma and Associated Factors in Saudi Arabia: A Systematic Review and Meta-Analysis

**DOI:** 10.1155/2018/8102527

**Published:** 2018-05-23

**Authors:** Shalam Mohamed Hussain, Syeda Ayesha Farhana, Sulaiman Mohammed Alnasser

**Affiliations:** ^1^Department of Pharmacology and Toxicology, Unaizah College of Pharmacy, Qassim University, Unaizah, Saudi Arabia; ^2^Department of Pharmaceutics, Unaizah College of Pharmacy, Qassim University, Unaizah, Saudi Arabia

## Abstract

**Background:**

Asthma is the most common reason for emergency visits to hospital and loss of productive hours. In Saudi Arabia, asthma affects more than 2 million people and majority of them have uncontrolled asthma with their quality of life adversely being impacted. It is well known that the prevalence of asthma has been increasing in many places around the world in the last few decades. The present review attempted to identify studies on asthma and associated factors in Saudi Arabian population and assess their time trends and regional variation.

**Methods:**

The titles and abstracts of retrieved articles were compared to delete duplication and irrelevant data. A data collection form was designed to extract several key components from selected articles like bibliographic information on the article, study population, and sample size. Extracted information was grouped appropriately for data analysis. Database search retrieved 71 articles. Applying inclusion and exclusion criteria, 40 articles were excluded and 31 qualified full articles were included for the review.

**Results:**

Among 31 retained studies, Riyadh had the highest publication output followed by Jeddah. Ten studies were based on ISAAC and 5 on non-ISAAC questionnaires while 5 studies were genetic studies conducted to unravel the genetic basis of asthma. Most of the studies were conducted on pediatric subjects of less than 16 years of age. Sample sizes ranged from less than 150 to more than 10000 with study settings being predominantly urban (22 studies). The pooled weighted prevalence rates of asthma, lifetime wheeze, and rhinitis were 14.3% (95% CI: 13.4–15.2), 16.5 (95% CI 15.5–17.4), and 21.4 (95% CI 20.5–22.3), respectively. An increase in asthma prevalence from 1990 to 2000 along with a stabilized or not so significant decline in the prevalence from 2010 to 2016 was observed.

**Conclusion:**

The prevalence of asthma varied in different regions without any disparity in prevalence in the rural and urban areas of Saudi Arabia. The understanding of genetic variability and recognition of risk factors in asthma patients can greatly help in individualizing the therapy for the management and control of asthma.

## 1. Introduction

Respiratory disorders are encountered with variable prevalence in different parts of the world and asthma is the most common among them. The prevalence of asthma has increased during the last decades in sync with increase in industrialization and modernization. As per World Health Organization (WHO), there are 235 million people worldwide suffering from asthma with approximately 383000 deaths due to asthma in 2015. Asthma impacts patients, their families, and the community as a whole in terms of lost work and school days, poor quality of life, frequent emergency department (ED) visits, and hospitalizations. Despite the advancements in the contemporary medicine, there are 40–70% of patients who have uncontrolled asthma [1, 2].

Saudi Arabia (SA) is geographically the fifth-largest state in Asia and second largest among Arab world. It is the only nation with both a red sea coast and a Persian Gulf coast on its sides. Its terrain consists of arid desert or barren land with varied geographical and climatic conditions, such as inland desert areas and dry environment (Riyadh, Hail), coastal humid environment (Jeddah, Jazan), industrial and nonindustrial, and urbanized and rural areas (Yanbu, Qassim, Najran). SA has a total population of 31.54 million with life expectancy of 73/76 years. The noncommunicable diseases (NCD) account for 78% of total deaths, out of which chronic respiratory diseases stand at 3%. The asthma affects more than 2 million Saudis, and recent studies suggest that majority of them have uncontrolled asthma with their quality of life adversely being impacted. These studies also attribute the prevalence of asthma to a host of factors including change in lifestyle, socioeconomic status, dietary habits and allergens, dust, tobacco smoke, sandstorms, and industrial and vehicular pollutants. According to Ministry of Health, SA, the prevalence of asthma ranges from 15 to 25% [1–4]. In the present study, review of all the research published in SA on asthma during 1990 to March 2017 was carried out with a special emphasis on reported regional variation in the prevalence, etiology, and pathogenesis. This review paves the way for policy makers and healthcare providers alike to frame guidelines for the proper management and treatment of asthma in lieu with current status of asthma.

## 2. Methods

### 2.1. Search Strategy

We carried out a systematic search of Medline, PubMed, and Google Scholar databases to identify original research (cross-sectional and population studies or case reports on asthma) conducted anywhere in Saudi Arabia from 1990 to 2017. We used the following key words (MeSH) for search strategy with asthma and Saudi Arabia as the main search concepts: prevalence, etiology, risk factors, quality of life, genetic basis, and demographic variations. However, searches were limited to articles published in English and studies involving humans.

### 2.2. Study Selection

The following screening criteria were used to select studies:

(1) Titles and abstracts of articles identified were carefully screened in the initial review for relevance to the topic.

(2) In the second review, articles were selected for inclusion based on relevant patient population (adults/children with physician-/questionnaire-diagnosed asthma), appropriate study design, and outcome measures.

(3) Studies were further checked for relevance to the topic.

### 2.3. Data Extraction and Analysis

**Figure 1:**
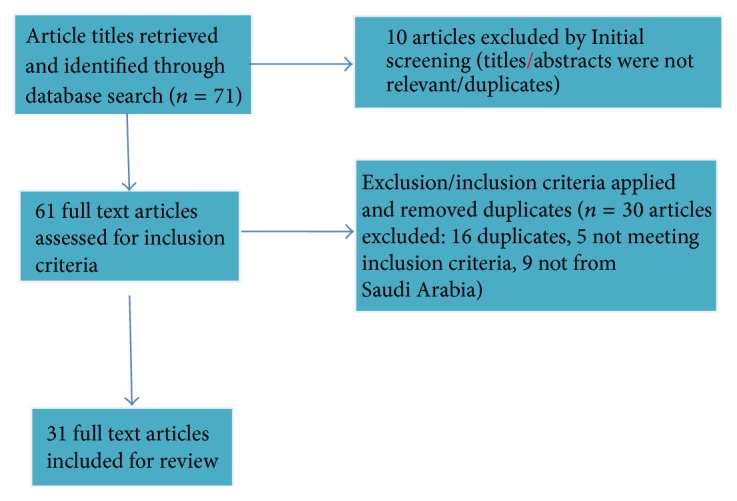
Systematic review flow diagram for how articles were screened, excluded, and included in the study.

The titles and abstracts of retrieved articles were compared to delete duplication and irrelevant data. Based on the mentioned eligibility criteria for the literature search, 71 articles were retrieved, 40 articles were excluded, and 31 qualified full articles were included for the review (Figure 1). A data collection form was designed to extract several key components from selected articles including bibliographic information on the article, study design characteristics, such as the study population and sample size, and diagnostic criteria used. We also extracted prevalence data including overall, sex-specific, age-specific rates and data on asthma diagnosis and/or its symptoms from each article. Extracted information was grouped appropriately for analysis.

### 2.4. Statistical Analysis

Forest plots with weighted mean and 95% confidence intervals (CIs) were generated with Neyeloff's method [5] and MS Excel (Microsoft Inc., Redmond, WA, USA). MS Excel was also used to calculate mean values and generate graphical data.

## 3. Results 

### 3.1. Study Characteristics

The 31 retained studies [6–36] covered different parts of Saudi Arabia. Riyadh alone contributed 11 studies followed by Jeddah with 5 studies. 15 studies reporting prevalence in the present review have used ISAAC (international study on asthma and allergy in children)/modified ISAAC/validated questionnaires as study instruments (Table 1). There are 5 genetic studies reported on asthma in Saudi asthma patients. Other included studies dealt with asthma control status and risk or triggering factors for asthma. Most of the studies were conducted on pediatric subjects of less than 16 years of age [6–18, 20, 22–36] and 3 studies conducted on adults [19, 21]. The studies enrolled school children as participants [6–20] and other studies were retrospective studies on asthma patients visiting hospital or PHC [21–36]. Sample sizes ranged from less than 150 to more than 10000, study settings were predominantly urban (27 studies), and 4 were mixed (both rural and urban) (Table 1).

### 3.2. Regional Variation in Prevalence of Asthma and Comorbidities

**Figure 2:**
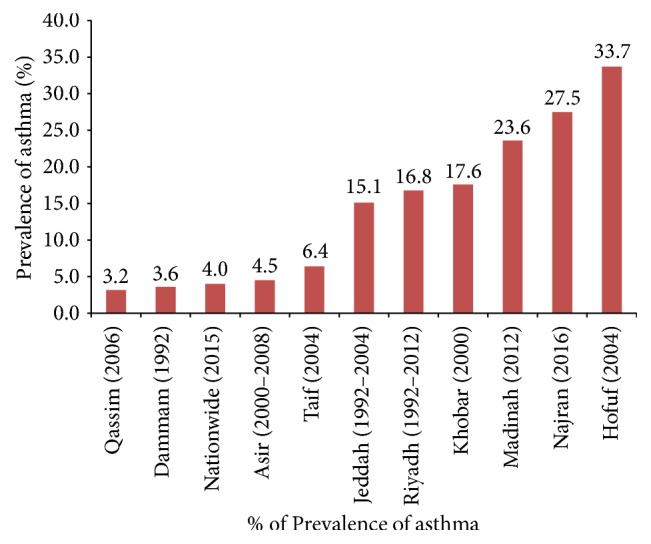
Regional asthma prevalence and publication period.

**Figure 3:**
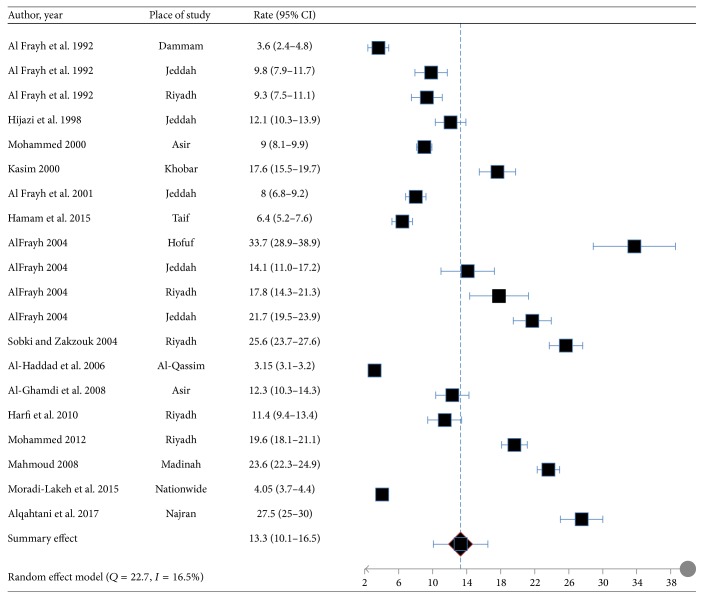
Forest plot for proportion of “asthma” and its 95% confidence interval in the prevalence studies.

**Figure 4:**
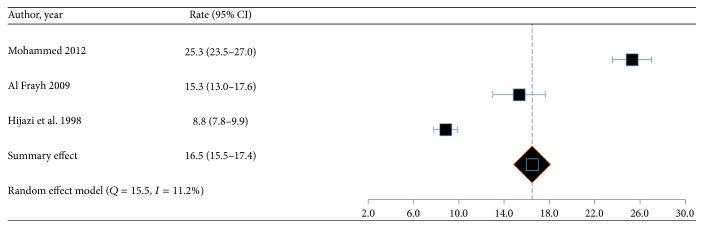
Forest plot for proportion of “lifetime wheeze” and its 95% confidence interval in the prevalence studies.

**Figure 5:**
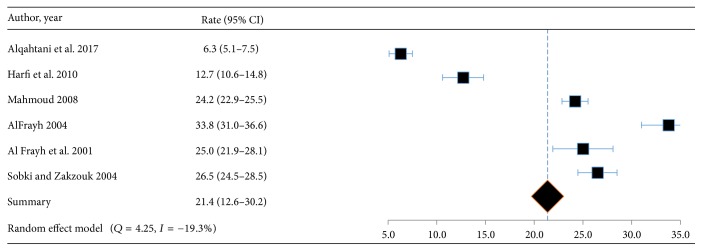
Forest plot for proportion of rhinitis (95% confidence interval) in the prevalence studies.

Among the regions of Saudi Arabia, the highest prevalence of asthma was found in Hofuf and the lowest in Qassim (Figure 2). Out of a total of 31 articles, 20 studies on asthma, lifetime wheeze, and rhinitis reported prevalence rates [6–20] which ranged from 3.5% to 27.5%, 8.8 to 25.3%, and 6.3 to 33.6%, respectively. The pooled weighted prevalence rates of asthma, lifetime wheeze, and rhinitis for these studies were 14.3% (95% CI: 13.4–15.2), 16.5 (95% CI: 15.5–17.4), and 21.4 (95% CI: 20.5–22.3), respectively (Figures 3–5).

The various individual studies were as follows: Al Frayh [6] conducted a multicity study among 3300 school children aged 7–12 years and found the prevalence of wheeze and hay fever in Jeddah (12.6%, 24%), Riyadh (11.9%, 17%), and Dammam (6.6%, 12.1%). Hijazi [7] reported a prevalence of 12.1%, 11.2%, 15.5%, and 12.2% in asthma, wheezing in last 12 month, cough, and rhinitis, respectively. Alshehri [8] randomly selected a sample of 4300 male school children aged 7 to 15 years in Abha and found the overall prevalence of asthma (9%), physician-diagnosed asthma (4%), exercise-induced asthma (4%), and wheeze in the past year (8%). Al Dawood [9] carried out a cross-sectional survey in Khobar with 1,482 school boys aged 6–15 years. He categorized his findings of prevalence rates as questionnaire-diagnosed asthma (QDA, 9.5%) and physician-diagnosed asthma (PDA, 8.1%).

Al Frayh [10] using ISAAC questionnaire reported an overall increase in the prevalence of asthma and allergic rhinitis from 8% to 23% and 20% to 25%, respectively, during the years 1986 to 1995 without any significant change in the prevalence of eczema during the same period. The same authors again in 2004 [11] described cumulative prevalence rates for asthma, allergic rhinitis and eczema at 21.7%, 33.8, and 36%, respectively, reporting a no significant change in asthma but an increase in allergic rhinitis and eczema, when compared to previous findings. Sobki and Zakzouk [12] using ISAAC questionnaire in Riyadh found the prevalence of 26.51% of allergic rhinitis, and 1/4th of whom were physician-diagnosed asthmatics. Hamam [13] enrolled 1700 school children and found the prevalence of asthma at 13.4%. Al-Haddad [14] enrolled 131190 general populations in 35 PHCCs and found the asthma prevalence at 3.15%. Al-Ghamdi [15] surveyed 1325 people aged 11+ years in Asir region and found the prevalence of bronchial asthma at 12.3%. Harfi [16] carried out a study in 1000 Saudi children aged 6–14 years using ISAAC in Riyadh and found the total prevalence of allergic rhinitis, asthma, eczema, food, and drug allergies at 12.7%, 11.4%, 5.6%, 1.75%, and 0.27%, respectively. In another study, Al Ghobain [17] using ISAAC questionnaire found the prevalence of lifetime wheeze, wheeze during the past 12 months, and physician-diagnosed asthma at 25.3%, 18.5%, and 19.6%, respectively. Nahhas [18] conducted a study in Madinah on school children using parents-filled ISAAC questionnaire and found the prevalence of eczema, rhinitis, and asthma at 10.3%, 24.2%, and 23.6%, respectively. Overall, 41.7% of children had symptoms suggestive of at least one allergic disorder and 7.4% of children had been diagnosed with rhinitis. Alqahtani [20] studied the prevalence and risk factors associated with allergic diseases in 1700 Saudi school children in Najran and reported the overall prevalence of physician-diagnosed asthma (27.5%), allergic rhinitis (6.3%), and atopic dermatitis (12.5%).

Moradi-Lakeh [19] published a nationwide cross-sectional study using a validated survey on 10,735 individuals aged ≥15 years and reported a cumulative prevalence of asthma of 4.05% (95% CI: 3.54–4.62%) with regional variations such as Jawf (1.82%); Tabuk, Hail, Riyadh (2.32–3.87%); Madinah, Asir, Najran (3.87–4.15%); Makkah, Qassim (4.15–4.36%); and Al Hudud, Ash Shamaliyah, Ash Sharqiyah (4.36–6.45%).

### 3.3. Time Trends in Asthma Prevalence

**Figure 6:**
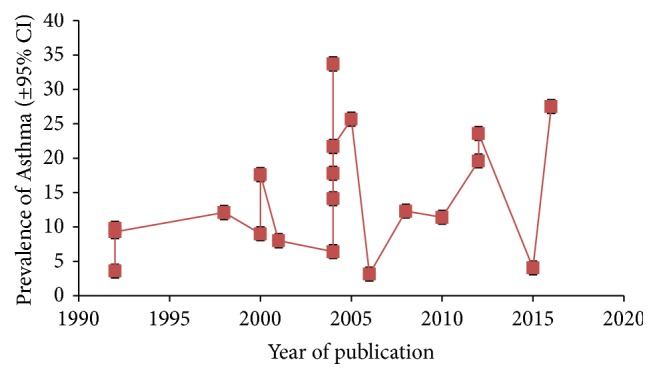
Asthma prevalence (95% confidence interval) over the years in Saudi Arabia.

**Figure 7:**
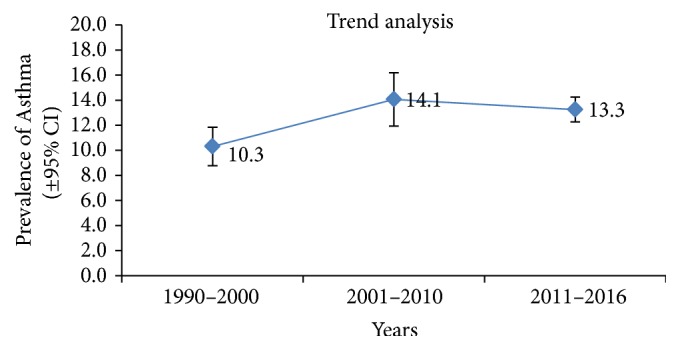
Time trends for asthma prevalence (95% confidence interval) from 1990 to 2016 in Saudi Arabia.

Al Frayh [6, 11] in their studies reported a sharp rise in the asthma prevalence from 1986 to 1990 and a stabilized behavior in 2004. In a study in 2010, Harfi [16] reported a declining trend in the prevalence of asthma when compared to previous studies (Figures 6 and 7). The available data internationally also suggest a global surge in asthma prevalence. The same trend may be playing in Saudi Arabia and more studies, however, may be needed to verify the claims.

### 3.4. Asthma in Urban and Rural Areas

The study settings for asthma studies were predominantly urban (22 studies [1–4, 6–16]) and 3 were mixed (both rural and urban). Hijazi in 1998 [7] studied the prevalence of asthma in Jeddah city and its adjacent rural areas using a cross-sectional questionnaire survey among 1,020 urban and 424 rural 12-year-old school children. They found 14.9% and 5.4% of asthma prevalence in urban and rural children, respectively. The prevalence of allergic symptoms was significantly higher in urban children than in rural [24]. Hamam in 2015 [13] reported that the majority of participants (96.1%) lived in Taif city while 3.9% lived in rural areas around Taif. The rate of asthma was higher in rural areas (16.7%) when compared to urban areas (12.4%) but this difference was insignificant (*P* > 0.05). Al Haddad [14] found the asthma prevalence rate between urban (3%) and rural (3.3%) areas among patients visiting PHCCs in Qassim region [37] without any significant difference in asthma management care. Asthma prevalence rate among girls (14.4%) was higher than boys (12.4%), but this was not statistically significant (*P* > 0.05%).

### 3.5. Asthma among Different Genders

Hijazi [7] found that males were more likely to have some respiratory symptoms and females had more eye and skin symptoms. However, Moradi-Lakeh [19] in 2015 found no statistical difference between age and sex subgroups in the prevalence of asthma in their study. Torchyan [21], in a study exploring the effect of gender on asthma quality of life (AQL) with a score of 1 to 7, indicated a poor to better quality of life. The study reported a mean AQL of 4.3 and 4.0 for males and females, respectively. This study also found that daily tobacco smoking had decreased AQL score in male whereas in females presence of a smoking family member had led to a corresponding lower AQL score. Conversely, a monthly household income of 25,000 or more Saudi riyals (SR) was associated with a better AQL among men and only a feeling of being employed exhibited a protective effect in females. BinSaeed [22] reported a significantly higher uncontrolled asthma in females (77.0%) than males (58.5%) and attributed this to higher levels of stress, daily tobacco smoking, and a monthly income less than 15000 SR. The same author in another study [25] explored the positive impact of knowledge of caregivers of asthmatic children on their asthma control. Albar [23] in a retrospective study on 194 asthma patients found no association between vitamin D deficiency and asthma control among males and females. Al Harthi [24] in Makkah reported an increasing trend in the prevalence of asthma from 16.6% in 2002 to 36.6% in 2006. The following triggering factors were found to have a positive relation on asthma in this study, family history (25%), change in weather (27.8%), dust (44.4%), exercise (22.2%), passive smoking (5.6%), and pets (16.7%). Males were more susceptible to asthma than females with dust being the most common risk factor triggering asthma.

### 3.6. Asthma and Its Genetic Basis

El Mouzan [26] found 52% and 59% positive parental consanguinity in asthmatic and control children, respectively, during a study involving 140 asthma children and 295 control children. Al Rubaish [27] found *β*2-AR gene polymorphisms at codon 16 and codon 27 in a case-control study. A significant difference was observed in genotype frequencies at codon 16 (Arg/Gly) between the asthmatic and control subjects (*P* < 0.05). Al Khayyat [28] also found T1 A/G and T2 G/A ADAM33 polymorphisms in Saudi asthmatic children with age and gender matched healthy Saudi citizens. Al Muhsen [29] found significantly higher IL-4 receptor alpha subunit (IL-4R*α*) and its single-nucleotide polymorphisms (SNPs), rs1805010 (I75V) and rs1801275 (Q576R), in Saudi asthma patients as compared to healthy subjects. Al Sulaimani [30] studied the genetic basis of asthma in Saudis and demonstrated three polymorphisms of CD14 promoter gene in asthma children.

### 3.7. Risk and Triggering Factors for Asthma

Abdalla [31] conducted a cross-sectional study in asthmatic children in Tabuk using a structured questionnaire to obtain information about frequency of exacerbations and possible triggering factors for asthma. They found that upper respiratory tract infection (85%) is the most common triggering factor, followed by dust, coldness, incense, smoke of woods, household chemicals, and passive smoking. However, no significant difference was observed in triggering factors between male and female children. Al Qahtani [20] found the following as potential environmental risk factors for asthma: presence of dogs in the house, being male, exposure to dense truck traffic in the living areas, use of wood as a cooking fuel, having a smoker family member, and performing vigorous exercise. Among dietary factors egg and vegetable intake were found to be significant risk factors for asthma. Similarly Hamam [13] found history of asthma in the family, smoking of family member, and exercise as risk factors for asthma. In another cross-sectional study using ISAAC, Hijazi [32] reported family history of asthma, atopy, eating at fast food outlets, and low intake of milk, vegetables, vitamin E, calcium, magnesium, sodium, and potassium as significant risk factors for asthma in a study involving asthma and healthy children in Jeddah and surrounding rural villages. Al Mazam and Mohamed [33] carried out a case-control study at the outpatient clinics of the two PHCCs (primary healthcare centers) in Bahrah and found that family history, allergic rhinitis, skin atopy, and recurrent respiratory tract infections are independent risk factors for asthma. In addition, they concluded specifically that the existence of a brick factory near the residences of studied population posed a significant risk factor for both the occurrence and severity of asthma, as these factories burnt fuel and tar to heat bricks polluting the surrounding environment and air. Al Binali [34] found that illiteracy, young age (<30 years), being female, and poor knowledge of asthma among mothers of asthmatic children are risk factors. Hamam [13] reported smoking family member, history of asthma in the family, and exercise as possible risk factors for asthma. Alangari [35] found no significant impact of sandstorm on acute asthma exacerbations in children. Al Ghamdi [15] surveyed 1325 people aged 11+ years registered at 2 PHCCs (1 at high altitude and 1 at sea level) in Asir region and found the significantly higher (19.5%) prevalence of bronchial asthma in people living at sea level when compared to people living at high altitude (6.9%). Authors also found that illiteracy, low income, use of coal and wood for heating, having a mud or tent house, lack of electricity inside dwellings, and presence of sheep are potential risk factors for asthma. Al Moamary [36] studied the factors responsible for refractory asthma in 74 cohorts of patients and found that two major comorbid conditions, allergic rhinitis (54.1%) and gastroesophageal reflux (33.8%), are associated with refractory asthma. The author reported that 97.3% of patients had at least one trigger factor for asthma and 86.4% of them had uncontrolled asthma.

## 4. Discussion

Asthma is an important health issue among children globally and a major concern for health authorities worldwide [38, 39]. It is a common heterogeneous chronic disorder of airways causing airflow obstruction with reversible and recurring symptoms due to bronchial hyperresponsiveness and underlying inflammation. The studies on asthma conducted in SA have dealt with its prevalence, etiology, risk factors, environmental and dietary influences, genetic basis of the disease, and quality of life of asthma patients [3, 4, 6]. The review included ISAAC and non-ISAAC studies, factors affecting asthma control, and studies understanding genetic basis of asthma in Saudi population. According to various researches an alarming rate of asthma is prevailing in Middle East with Saudi Arabia taking the lead with a rate of 24% of the population suffering from asthma followed by Qatar, Kuwait, United Arab Emirates, and Oman. Its impact is manifested in patients, their families, and community as a whole.

### 4.1. Regional Variation in Prevalence of Asthma and Comorbidities

Saudi Arabia represents a unique country with diverse climatic conditions and topographical features. The highest prevalence of asthma was reported from Hofuf, Madinah, and Najran. In contrast the lowest prevalence rates were reported from studies in Qassim, Dammam, and Taif. The studies attributed high rate of prevalence to a host of factors including change in lifestyle; spread of urbanization; socioeconomic status; dietary habits; higher exposure to indoor animals, allergens, dust, tobacco smoke, sandstorms, and industrial and vehicular pollutants; and location of residences. However, more studies may be required to reach a conclusion regarding the climatic influence on asthma prevalence. The most common comorbidities reported with asthma were wheeze, rhinitis, and cough [6–11].

### 4.2. Asthma in Urban and Rural Areas

Studies have suggested that there is more asthma in urban than in rural areas in many parts of the world. Early studies from Africa (South Africa, Ethiopia, Kenya, and Ghana) reported that populations living in rural areas (i.e., not exposed to the effects of an urban or western lifestyle) experienced a very low burden of allergic disease, and a traditional rural way of living provided a possible protective cover. Similar studies from Asia (China, Japan, Korea, India, and Saudi Arabia) confirmed the urban-rural gradient due to exposure to different allergens, air pollution, affluence, and diet in the development of asthma and allergy [37, 40]. Hijazi [7] found more asthma and allergic symptoms prevalence in urban than rural children. In contrast Hamam [13] reported a higher rate of asthma in rural areas when compared to urban, but this difference was not statistically significant. Similarly Al Haddad [14] found the asthma prevalence rate between urban and rural areas of Al-Qassim insignificant. Though higher asthma prevalence does not appear to be related to urbanization per se, it could be associated with the increase of population susceptibility rather than changes in exposure to allergens [37, 41]. Some investigators reported high asthma prevalence rate among children with passive smoking and a history of asthma in the family. It also varied from region to region due to the variability of extrinsic allergens that might play a major role in infection of susceptible children [4]. A study determined the possible environmental and dietary determinants of asthma among school-aged children in Saudi Arabia and found that dense truck traffic on the street, using wood as a cooking fuel, vigorous exercise, consuming eggs, vegetables, and Bermuda grass likely increase the risk for asthma [21].In the present review no significant difference in the prevalence of asthma between rural and urban regions was retrieved and other concrete factors may be retrieved by focussing on the etiological aspects of the disease in these areas.

### 4.3. Risk and Triggering Factors for Asthma

Many studies in Saudi Arabia found that upper respiratory tract infections, gastroesophageal reflux, dust, coldness, incense, smoke of woods, household chemicals, and passive smoking are common triggering factors and that smoking of family member and history of asthma in the family, exercise, atopy, eating at fast food outlets, low intake of milk, vegetables, and vitamins, and allergic rhinitis are triggering factors for asthma [13, 15, 20, 31–33]. Al Mazam and Mohamed [33] also reported in their investigation that the presence of brick factory near the residences of their studied population posed a significant risk factor for asthma. Al Binali [34] concluded that illiteracy, young age (<30 years), being female, and poor knowledge of asthma among mothers of asthmatic children are factors that can also pose risk. These results also are in compliance with studies that determined that risk factors are contributing factors for asthma [38, 39, 42]. Al Ghamdi [15] also reported that people living at sea level had higher prevalence of asthma than those living at high altitude. Al Mazam and Mohamed [33] opined that risk factors were responsible for refractory asthma. Similarly, BinSaeed [22] observed that asthma control decreased with reduced household incomes and sharing of a bedroom by asthma patients with siblings. Overall many factors that can contribute to asthma or trigger acute attacks of asthma among patients should be addressed when individualizing the management therapy for optimum outcome.

### 4.4. Asthma and Its Genetic Basis

Asthma is believed to be a complex genetic disease resulting from the interplay of multiple genes, environmental factors, and dietary or life style modifications. Reports have indicated that the first degree relatives of asthmatic individuals are at increased risk of asthma when compared to general population [39]. Heredity and phenotypic variances also may account for development of asthma. El Mouzan [26] found a positive parental consanguinity in asthmatic patients. Studies found gene polymorphisms in *β*2-adrenergic receptors and T1 A/G and T2 G/A ADAM33 in Saudi asthmatic patients [27, 28]. Other studies reported significant single-nucleotide polymorphisms (SNPs), rs1805010 (I75V) and rs1801275 (Q576R), in IL-4 receptor alpha subunit (IL-4R*α*) and CD14 promoter gene [29, 30]. These studies indicate that asthma can aggregate within families that are autosomal recessive with specific gene variants. Further attempts may be made to explore any mutation in a major gene that can cause asthma. The approach can also help in finding inducer or inhibitor candidate genes for asthma in Saudi population. These genomic data and tools can help to uncover the etiology that can drive individualized management plan.

### 4.5. Time Trends in Asthma Prevalence

The present review of the published literature observed a sharp rise in the asthma prevalence from 1990 to 2000 with a stabilized rate during the period 2000 to 2010 and a slightly declining trend there onwards in the prevalence of asthma [6, 11, 16]. The available data internationally suggest a global surge in asthma prevalence and the sense of optimism that asthma prevalence may be declining appears unfounded [2, 4]. In a review [38], it was shown that before 1990 asthma mortality was common in Netherlands but dramatically dropped when guidelines were framed for use of inhaled steroids in respiratory symptoms and asthma. Similarly a study in Finland reported 80% decrease in hospitalization and mortality following a significant increase in use of inhaled steroids for asthma. Asthma prevalence in Saudi Arabia may also be attributed to measures of healthcare taken to improve the quality of care in the treatment. However more studies may be needed to verify the claims that prevalence is declining.

### 4.6. Strengths and Limitations of This Study

The main strengths of this study are the comprehensiveness of the searches involving studies conducted all over Saudi Arabia. The resulting asthma trends presented for Saudi Arabia in the present review are most comprehensive till date and we hope this will be of considerable benefit to healthcare planners locally. There are, however, a number of limitations that need to be considered. The included studies did not have a uniformity in questionnaire, symptom classification, and studied triggering factors. Also there was a wide variation in study samples used. It is also limited by the quality of the included studies as well as the unavailability of some relevant data for comparison and analyses. It is also to be noted that the long period of study selected saw an unprecedented growth and development in the region leading to overall changes in the monetary and life style of its populace.

## 5. Conclusion

The prevalence of asthma in different regions of Saudi Arabia varied with the highest being reported in Hofuf and the lowest in Qassim without any disparity of prevalence in rural and urban areas. A declining trend is observed nationally, though not a significant one. The common triggering factors found are upper respiratory tract infections, dust, coldness, household chemicals, passive smoking, and allergic rhinitis. The asthma pathogenesis reviewed in the present study had a genetic variability on Saudi population. The understanding of genetic variability together with recognition of specific factors influencing interactions between genetic and environmental components can greatly help in individualizing the therapy for the management and control of asthma.

## Figures and Tables

**Table 1:** Characteristics of studies conducted on asthma in different regions of Saudi Arabia.

	Number of studies
*Cities*	
Riyadh	11
Jeddah	5
Asir	3
Khobar	2
Taif	2
Tabuk	2
Madinah	1
Makkah	1
Najran	1
Qassim	1
Dammam	1
Hofuf	1
Bahra	1
Nationwide	1
*Sample size *	
>10000	4
>3000	4
<2000	8
<1000	15
*Study setting *	
Rural	0
Urban	27
Mixed	4
*Study instrument *	
ISAAC	9
Non ISAAC	6
Case report	11
Genomic study	5
